# A discourse analysis of Ebola in South African newspapers (2014–2015)

**DOI:** 10.1177/0081246319868656

**Published:** 2019-08-13

**Authors:** Prevan Moodley, Schvaughn Sandrine Lesage

**Affiliations:** Department of Psychology, University of Johannesburg, South Africa

**Keywords:** Discourse analysis, Ebola, news reports, South Africa, virus, West Africa

## Abstract

The 2014 Ebola outbreak in three African states transformed the virus into a social reality in which media representations contributed to globalised hysteria and had rhetorical effects. This study investigated representations of the Ebola virus/disease in South African news reports (March 2014–June 2015). Four discourses were found to operate within the globalised social context: threat to humanity, predation, invasion, and conspiracy. The South African reportage framed Ebola as a predator and criminal rather than using stock warfare imagery. Representations indicated alignment with phobic high-income countries and colonial hegemony.

The fear elicited in press coverage of the 2014 Ebola outbreak led to public reactions being dubbed ‘Fearbola’ (James, 2014). Sensationalist discourse emerged, particularly through dire news headlines and photographs, both in high-income countries and the affected countries (Halsey, 2016). Halsey (2016) states that the affected regions’ local press often contained faulty information that contributed to social constructions framed by fear. The focus of the current study is on how the 2014 Ebola outbreak was represented in South Africa, specifically on how the virus or disease, rather than its management, was represented in news reports.

Emotive engagement with Ebola in the media illustrates how medical discourse became political and ideological, as was shown in Kamalu’s (2016) critical discourse analysis of comments posted on blogs, discussion forums, twitter, and Facebook (July–October 2014). Focussing on these online posts, which represented public meanings in Nigeria, Kamalu (2016) found that posts actively evaluated government reaction and policy, circulated ethnic and racist discourse, and reinforced notions of Ebola both as a form of terrorism and othering. Online media thus played a role in public circulation of fear and hysteria, and also contributed to (improper) psychosocial management of affected persons. To demonstrate this, Mondragon, de Montes and Valencia (2017) showed that laypersons’ social representations depicted Ebola as being definitively African (particularly in being linked to poverty), along with portrayals of dread about entering the affected countries and ‘backward’ Africa lacking competence to manage the disease. Localised social support for and stigma towards survivors added to the global fears originating in high-income countries, detracting from public education, as well as from the political and economic challenges in the affected regions (Foster & Dashwood, 2014). The fear, which implicated stigma, showed that implementing just a clinical response to patients was insufficient (as the human immunodeficiency virus [HIV] crisis warned society previously) because psychosocial care was needed to help both stigmatised patients and their caregivers (Davtyan, Brown, & Folayan, 2014; van Bortel et al., 2016). Survivors were stigmatised through a contagion discourse because they were denied jobs by potential employers and refused treatment by health workers (Mayhew, 2016).

The social problem that results from fear is one of positioning particular people as dangerous others, a common response in the history of epidemics. Othering is fuelled by fear, and the dehumanising effect, which may ease the process of othering, leads to the blaming of infected persons and governments who fail to control the spread of the disease. Such representations, with reference to Ebola, depicted the African as the ‘Other’ and therefore, inferior, further progressing to a discourse of hope for containing the infection through the superlative promises of biomedicine being delivered from the industrialised world (Washer, 2004).

Othering representations, however, have shifted from downward othering of marginalised groups towards upward othering of governments and organisations such as the World Health Organization (WHO) (Joffe, 2011). For the Ebola crisis, such othering led to social representations of conspiracy. According to social psychology explanations, the ingroup (the affected group in this case) attributes the calamity to an act of government or to an outgroup because their sacred values are threatened (Franks, Bangerter, & Bauer, 2013).

Nevertheless, othering is appropriated within other discourses. In the discourse of hope (Washer, 2004), the risk of infection remains relegated to ‘primitive’ peoples, making this the West’s way of talking so that it does not consider itself to be at risk as long as its borders are not transgressed. As conceptualised by Aaltola (2012), Western lay people usually watch a scenario unfolding elsewhere and judge performers on a foreign disease stage. This reinforces the ‘us’ and ‘other’ distinction. The affected regions get viewed in Western newspaper reports through colonialist images of Africa as a jungle where people eat monkeys and fruit bats, and have cultural practices that promote disease transmission (Abeysinghe, 2016). Olivier (2007) considers that any uncontrolled constituent of nature such as a virus (particularly when associated with an African jungle) represents a threat to humanity and civilisation, and is relegated to an ‘abject’ position. This threatens Western capitalistic society’s notion of taming nature and, in so doing, shows up the precariousness of a technological society whose survival is based on the idea that nature is controllable.

Historically, images have been used to construct particular versions of diseases, as Sontag’s classical theorising has shown. Predominant explanations have used war metaphors. These have included notions of battle, combating of infection, counterattack, invasion, and fight (Sontag, 2003). Metaphors function as a conduit to disseminate scientific knowledge to nonexperts, but they have discursive and sociopolitical consequences (Larson, Nerlich, & Wallis, 2005), contributing to particular media framings of disease, such as those of war or a plague in the case of Ebola (Vellek, 2016). Vellek’s (2016) deductive content analysis shows that such images in reports in wide-reaching newspapers in the US, UK and Singapore located Ebola within a mutation-contagion frame. However, using war metaphors is a choice made by journalists. As Larson et al. (2005) have shown, UK newspapers *avoided* war metaphors in reports on severe acute respiratory syndrome (SARS), an emerging infectious disease (EID), just like Ebola.

Even though war images add newsworthiness (Larson et al., 2005), the militarisation of illness creates stigma for those infected. Sontag (2003) asserted that discourse of war and battle should be avoided because it promotes dread and stigma. Popular culture and media have also extended the war metaphor to other-worldly battles of science fiction. Diseases such as acquired immune deficiency syndrome (AIDS) were likened to alien invaders and envisioned as the alien ‘other’ (Sontag, 2003). The imagery of outer space further reinforces the otherness through visuals of hazmat suits that get likened to space suits and thus to science fiction (Joffe & Haarhoff, 2002).

Common images about social, psychological, and national threats have been used in Ebola discourse. Predominantly, the Ebola virus, based on the outbreaks occurring until 2001, was subjected to a rhetorical construction of predation (Weldon, 2001a). Weldon argued that, in comparison to other deadly viruses, Ebola does not kill millions of people and representations of the epidemic were based on misperceptions and, specifically, on predation images derived from a 1994 text, *The Hot Zone* (Preston as cited in Weldon, 2001a, 2001b). Weldon’s (2001a) analysis of the predatory discourse showed that ‘the issue with Ebola involves a rhetorical construction, in addition to a social construction’ (p. 8). The discursive construction of Ebola functioned to detract responsibility from the problem of compromised medical practice for managing Ebola. This shift, through personifying the virus that led to fear being elicited by this construction, removed focus away from the poor health systems and practices of the affected areas. Movies and books, which capitalised on this image at that time, further shifted agency away from humans responsible for the spread (Weldon, 2001a). Images of war, fire, and mythical potency contributed to this predatory discourse. Weldon (2001a) argued that these were exaggerations because of the epidemiological fact that needle sharing at clinics in Zaire and Sudan led to fatalities in the earlier epidemics. In other words, governmental systems and human agency threatened the broader-accepted belief system that Western medicine was infallible. As one epidemiologist and microbiologist also pointed out, the clinical picture presented in Preston’s book is inaccurate (T. C. Smith, 2014).

Weldon (2001a) further speaks of Ebola’s representation within conspiracy discourses because of the global threat of bioweapons. Besides state or US conspiracy, human blood was positioned as a coagent or as being in conspiracy with the virus. Through this discourse, the sentient virus instructs the blood what to do, transforming Ebola into a mythical entity that displaces the human medical practitioner as true agent of transmission in the epidemics that occurred until 2001. Whereas an ‘objective’ account would point out human mistakes and the health-practitioner carelessness, Weldon (2001a) attributes the dominating predatory discourse to Preston’s classic, purportedly nonfiction text.

Research into analyses of representations of the 2014 outbreak, even if it recognised the sensationalism effect (James, 2014) or used systematic empirical methods (e.g., Paul, Mahajan, & Sahoo, 2016; S. Smith & Smith, 2016), did not include in-depth analyses of the ideological and discursive portrayal of the disease, a gap addressed in this article. S. Smith and Smith’s (2016) quantitative content analysis of four Nigerian newspapers simply coded for reporting genre (e.g., editorial) and type of content (e.g., death rates) in 1625 articles; and Paul et al. (2016), despite confirming the globalisation of fear, presented frequency counts as opposed to showing how the fear was discursively constructed in their quantitative content analysis of three newspaper publications in India. In this article, we therefore aim to illustrate how fear was the effect of particular representations that emanate from cultural and metaphorical content in news reports in the 2014 Ebola epidemic. Put differently, we aimed to identify discourses that created a sociocultural reality about Ebola.

## Method

### Sample

This final or refined sample consisted of 113 news reports. These were labelled systematically for source tracking.

### Procedure

We typed in the keywords ‘EBOLA’ and ‘SOUTH AFRICA’ into the online news database South African Bibliographic and Information Network (Sabinet), where South African newspaper articles are archived. The date range was restricted from March 2014, the date when the WHO officially announced the Ebola outbreak, to June 2015. June was chosen as the end-date because that was when data analysis commenced, despite the ongoing occurrence of Ebola beyond this date. Even so, news coverage of Ebola became saturated and tailed off in November 2014.

The keyword search identified 145 articles in full text (pdf) format. After reading all articles, a manual search excluded those that did not relate directly to the Ebola virus. We removed 34 articles because they did not discuss the Ebola virus itself. The excluded articles either addressed the management of the epidemic or did not refer to explicit cultural representations of the virus. We were interested in how the disease itself had taken on a particular social and metaphorical reality.

### Ethical considerations

Because we used archived published press reports, no ethical clearance was required. The data are in a public database domain. Nevertheless, in the interpretation of data, we ensured we were transparent with the quoted extracts and made interpretations supported by textual evidence.

### Data analysis

We used Parker’s (1992) guidelines for analysis. Keywords and phrases in the reports were subjected to a process of free association. In other words, shared social meanings were listed by free associating to words and phrases, but which were relevant to the research question. This led to describing how the Ebola virus/disease was understood in the texts, making way for identifying discourses or the ways in which the disease/virus was constructed. Both researchers critically reflected on the meanings arising from associations to ascertain if they contained unduly idiosyncratic or overpersonalised projections, and they validated each others’ inputs throughout analysis. Simply, they discussed associations and reached consensus by focusing on the relevance of meanings to the research question. Because the meanings arose from the social worlds and culturally consensual realities of the researchers as South Africans who wished to interrogate the representations of diseases, but who were also often sympathetic to African disasters and human loss, they acknowledge that, in line with discursive practice, their shared subjectivities are instrumental in making interpretations following a poststructuralist approach as opposed to aiming for an objectivist and realist encounter with the texts.

## Results

Four discourses have been selected for this article. These focus on social discourse, even though the expected medical discourse was also often represented in the texts. The medical discourse consisted of facts about the clinical picture and the transmission of the virus, in contrast to our focus on how the disease achieved a social and cultural reality, a version that contributed to barriers in better addressing the epidemic. We thus exclude the medical discourse in the reporting of our findings.

### Threat to humanity discourse

The texts represented the Ebola virus as a threat to humankind, transforming the epidemic into a pandemic. Manifested through words such as ‘epidemic’, ‘pandemic’, ‘threat’, and ‘devastating apocalyptic epidemic’, Ebola induced global panic and fear:

Senegalese President Macky Sall grabbed that ball and ran with it, chiming in that: Ebola is not an African disease. It is necessary to confront Ebola as a threat to humanity. (Fabricius, 2014)

The Ebola outbreak in West Africa is fast developing into the devastating apocalyptic epidemic mankind has feared for so long. (‘Help Needed in Ebola Crisis’, 2014)

The reportage assumed that borders were permeable. The 2015 Ebola epidemic moved from being a threat to specific populations (affected countries) to a collective threat due to globalisation. This was noted within the image of crossing of national borders: ‘The Ebola pandemic is a humanitarian crisis of tragic proportions . . . extending beyond these countries’ borders’ (Serrao, 2014). Regarding the threat to South Africa, reportage aimed to reassure citizens: ‘South Africa is capable of dealing with and containing Ebola if the viral disease reaches our shores. This was the assurance from government as fears of a global Ebola outbreak spread across the world’ (Mapumulo, 2014).

### Predatory discourse

The virus was referred to as either a human or animal predator. Personifications such as ‘killer’ and ‘agent’ implied murder. This suggests vigilance because reader–victims are unaware who and when this *criminal* will target next:

The outbreak of Ebola in West Africa has seen, and rightfully so, a global response to a ruthless killer that science is yet to work out how to curb. (‘Educate Nation on Ebola’, 2014)

It is a scenario that science fiction writers often toy with – an infectious agent, lurking in the shadows, suddenly pouncing when an opportunity presents itself and wreaking havoc. (‘Nature’s Assailants’, 2014)

[Ebola has] slain more than all previous outbreaks put together. (Vallely, 2014)

Headlines used the discourse of predation:

Nature’s assailants [Headline]. (‘Nature’s Assailants’, 2014)

Ebola steals the human touch [Headline]. (Patta, 2014)

Timeline of a killer on the rampage [Headline]. (‘Timeline of a Killer on the Rampage’, 2014)

These associations to crime (‘steals’, ‘killer’, ‘lurking’, ‘slain’) prefigured a forensic science narrative, so that the predatory discourse presented the movements of the virus as subject to surveillance and crime management. Using crime associations to the virus, particularly within headlines, adds to newsworthiness and befits the news genre that needs to attract readership.

Lethality is made more frightening when the personification crosses over into the animal world. The press reports showed that evolved human rationality, now attributed to an animal, made the virus even more unpredictable. Images such as ‘molecular shark’ (‘Guinea Races to Stop Growing Ebola Epidemic’, 2014), ‘killer’, and ‘infectious agent’ combine ideas about human and animal predators. The imagery of hunting and stalking represents the virus as purposeful agents intending to eliminate the human race. Thus, Ebola was accorded primitive but instinctive intentionality. Like an animal hunting in wild Africa, the Ebola virus was represented as picking out vulnerable populations first and spreading thereafter to those who assist and care for affected groups.

### Discourse of invasion

Ebola was portrayed as an invading entity moving from country to country, leaving death and devastation in its wake. This was conveyed through the use of words and phrases such as ‘sweeps’, ‘growing’, ‘fire’, ‘inferno’, and ‘raging out of control’. These images portrayed Ebola as unstoppable, permeating borders at will:

The government of Guinea has raised the death toll in the Ebola epidemic raging through its southern forests and capital to 95. (‘Nature’s Assailants’, 2014)

The Ebola outbreak has become a tale of two diseases: one quickly controlled and cured in the case of two US aid workers, and another that is raging out of control in poorly prepared African countries whose infrastructure is either basic or nonexistent. (Patta, 2014)

The blame for this spread is distributed both to the industrialised, wealthy nations and to poor African countries. The West is implied as not doing enough for Africans, mobilising quickly and effectively only when their own citizens are infected. In contrast, the poor African countries are seen as kindling the virus because their compromised infrastructure further spreads the disease. This leads to blaming the affected countries and their populace, creating an ‘us’ (enlightened and civilised persons) versus ‘them’ (primitive Africans) mentality.

### Conspiracy discourse

The conspiracy discourse that constructed Ebola as a fabrication occurred through reports using words such as ‘lie’, ‘invented’, ‘fake’, and ‘hoax’. As a conspiracy, the virus was portrayed as arising in the racist West and directed at primitive Africans. The primitive person was positioned by the developed world as having broken taboos and ‘civilized’ norms (e.g., eating bushmeat). Meanwhile, marginalised groups in Africa blamed the West for bringing the virus to Africa and unleashing it on them:

Academic Jules Evariste Toa said that in some rural communities, people continue to eat bush meat and tell themselves that ebola is a virus invented by white men to decimate the African population. (‘Caught Between Rumours, Hard Facts’ 2014)

One factor was that it is difficult to convince communities to go to a clinic when they are sick, because they believe they will die if they enter a clinic. ‘Many also believe the virus is fake or that we brought it here’, he [doctor working for MSF] said. (Coetzee, 2014)

And the conspiracy theory drumbeat is a little scarier when it comes from people who would seem to carry some more legitimacy – like the Liberian-born US professor who recently wrote in a Liberian newspaper that the Ebola outbreak was the result of US bioterrorism experiments. (‘Spare Us These Idiots’, 2014)

The second type of conspiracy theory is directed not at the outside Western world, but at activities within the affected countries’ borders, such as governments being blamed for lying in order to raise funds and so control them:

Cases were isolated at the public clinic and preparations made for transferring them to Kenema’s Lassa fever ward. This is when the rumours started spreading to Kono. Outside our clinic, a woman yelled, ‘Ebola is a lie! They’re sending people to Kenema to die!’ (Frankfurter, 2014)

In Monrovia’s largest slum, West Point, angry protesters broke into an isolation ward this week, chanting ‘Ebola is a hoax’ and accusing President Ellen Johnson Sirleaf of using the disease as a scam to raise money. (Patta, 2014)

[T]he Ebola outbreak is attracting conspiracy theories, with claims ranging from the unsubstantiated to the downright crazy . . . as the Washington Post reported, singer Chris Brown informed his Twitter followers that Ebola was some kind of population control plot. (‘Spare Us These Idiots’, 2014)

The discourse of conspiracy, through the view that Ebola is fabricated, detracts from a community’s responsibility to address the seriousness of the disease. The discourse, for those who believe in its veracity, allows ‘primitive’ Africans to believe they are safe and keep their integrity intact, given the history of colonialism and high-income countries’ hegemony over cure of disease. This discourse thus serves as a barrier to medical management. As a psychological defence towards colonial oppression, conspiracy beliefs lead to an increase in political tension, both within international relations and the affected countries.

## Discussion

The threat to humanity discourse found in the current study is typical of media representations of Ebola outbreaks that occurred until 2001 (Weldon, 2001b). The apocalyptic references in our findings were also invoked in nurses’ utterances about Ebola (Broom & Broom, 2017). Such discourse, however, is not limited to Ebola: In an analysis of how British newspapers portrayed a particular agricultural threat, Larson et al. (2005) found apocalyptic and providential references, with these metaphors complementing warlike responses to disease. However, the threat to humanity discourse can have an enabling outcome to motivate addressing the pandemic. Weldon (2001b) maintained that globalisation rhetoric was deployed to get the virus recognised, leading to renewed medical research. To get help to those most affected because of their less sophisticated health care systems, representation work must resort to personalisation of the threat, even though the discourse remains problematic.

Key to the threat to humanity discourse is the stock image of spread that is characteristic of pandemic risk. Pandemics imply ‘movement, directionality and contagious spread’; boundaries become porous, leading to zones cordoned off for containment (Aaltola, 2012, p. 668). This discourse was also found in Vellek’s (2016) content analysis of newspaper reports of Ebola as a global threat, one made possible by easier travel options in the modern world. However, this discourse, when used in the earlier outbreaks in Zaire and Sudan, had the effect of protecting individual governments because it sidestepped the real factors that led to fatalities, that is, the economic and systemic constraints reflected in staff not being trained or enough supplies not being provided (Garrettas cited in Weldon, 2001a). Kamradt-Scott (2016), therefore, in explaining the WHO’s reform of global health security – although late for West Africa – comments that the affected countries’ governments should have been more proactive by asking for help because of the global risks. Thus, the effect of the threat to humanity discourse is to detract from national governments’ responsibilities and failures, allocating passivity and even invisibility to their actions and roles while uncontrolled agency is granted to the virus. The uncontrolled agency is also figured into the discourse of invasion which reveals the process that unfolds with graphic descriptions of territorial uncontainment. In other words, the threat to humanity discourse, which rests on the universal globalised fear, is the outer layer of the sinister (unseen) process of invasion.

The discourse of invasion was similarly found in the theme of border control in Abeysinghe’s (2016) research of newspaper articles of the 2014 Ebola epidemic in three countries. Newspaper narratives of three Western nations (Australia, US, UK) focussed on Ebola as a problem for the West and on how political governance could manage the Ebola invasion. Abeysinghe’s (2016) study of news reports showed that Ebola became written about not as an African problem or global problem, but as a problem for once safe and enclosed countries – a discourse also taken up by South African texts in our sample. The discourse of invasion by a force of nature feeds into political and psychological fears. Consequently, Australian health professionals experienced anxiety and were faced with moral challenges because they had to decide ‘*how* benevolent they were, *how* much they would sacrifice, and the extent of their care’ (Broom & Broom, 2017, p. 213, italics in the original). Furthermore, invasion discourse is key to militaristic metaphoricity. As Larson and colleagues (2005) – referring to invasive species – explain, news reports, when allocating agency to an entity, are able to justify militaristic and nationalistic responses.

The discourses of threat to humanity and invasion are also given an organic face and figuration, either human or animal, via the discourse of predation. The predation discourse is in line with positioning the virus as abject, through which the menacing potential of nature threatens the technophiliac illusion that a virus can be controlled by the achievements of Western civilisation (Olivier, 2007). Predatory images provoke fear about dangers of the natural world that need taming and control (Olivier, 2007). The social impact implied in the image of a killer, for example, and particularly when linked to a particular geographical location, is for othering to be increased (Larson et al., 2005). Weldon’s (2001b) analytic work about the representations of Ebola as an invisible predatorial virus in nonfiction accounts, such as in Preston’s *The Hot Zone*, shows that this imagery transforms the Ebola hemorrhagic fever into a legend. When these images move from books to the news media, blurring occurs between the line of purportedly objective news representations and those descriptions aiming for literary and emotive value.

Similarly, Ungar (1998) identifies the use of words such as ‘assailant’ and ‘killer’ in newspaper articles describing Ebola, but within military discourse. The most widely used frame discovered in Vellek’s (2016) study even subsumes notions of a ‘killer’ alongside war and plague metaphors, rather than considering the killer from within a predatory model. The current study found greater reliance on the predatory model compared to militaristic images of the disease. This could be attributed to sampling because we selected news reports about the virus itself, rather than about how the disease is being managed.

Nevertheless, predatory images have rhetorical effects. Weldon (2001a) found that anthropomorphising of the virus through a predatory model downplays ‘the extent of human involvement in the transmission of disease in favor of a much more titillating story of a predatorial virus’ (p. 18). Such a discursive representation is dangerous because it adds to ‘ignorance regarding our biological relationship with our world, especially as it is mediated by environmental explorations, coupled with scientific and medical interventions’ (Weldon, 2001b, p. 293). The global public, as audience of this myth, is distracted from their role in taking responsibility for the spread and prevention of the disease (Weldon, 2001b).

Another way of detracting from political and national responsibilities for managing disease outbreaks, whether as an intrapsychic coping strategy or a delineation of ingroup–outgroup distinctions and politics, was illustrated by the finding of the discourse of conspiracy. Stigmatised groups redefine how they are represented (e.g., as unsanitary) through the use of conspiracy theories (Joffe, 2011). Washer (2011) asserts that the aim of conspiracy theories is to direct blame back at the West, thereby challenging dominant medical discourses. Our findings showed two types of conspiracy theories, based on whether agency was directed either to phobic high-income countries or within the affected countries. Conspiracy notions led to an uncoordinated and slow response because, close to civil war zones, people in the outbreak area distrusted the government. Infected people did not access treatment facilities (representing the untrustworthy government), and they died at home: This added to understaffed facilities not managing contagious dead bodies, resorting to sending the bodies back to their homes (Brown, Arkell, & Rokadiya, 2015). The conspiracy theme, represented in movies, mobilises global action (Weldon, 2001a). Thus, journalists’ quoting of people’s conspiracy ideas accentuates global attention. Conspiracy theories also allow people to cope with collective trauma – particularly when attributing causes to outgroups and in doing so, they also problematise the dominant discourses (Franks et al., 2013) which, in South African press reports, related either to medical understandings or predatory images.

A limitation of this study is that, because Sabinet relies on manual processing, it is possible that articles may have been mistagged and therefore did not appear in the search results. In addition, there may have been newspapers that were absent from the database. That the outbreak occurred 3 years ago does not imply the current study is delayed or irrelevant. The 2013–2016 Ebola outbreak was the largest, with approximately 28,639 cases (Centers for Disease Control and Prevention [CDC], 2016). In comparison, approximately 2,427 cases were reported in all outbreaks before 2013 (CDC, 2016). Fatalities from the 2013 outbreak, as well as the media representations, point to lessons. The fear discourse spread worldwide, unlike the current 2018–2019 outbreak in the Democratic Republic of Congo (DRC) that has approximately 1,082 cases since August 2018 (WHO, Regional Office for Africa, 2019) and no similar global hysteria. The DRC outbreak has been difficult to manage partly because of violence stemming from the community’s mistrust of Western medicine and medical staff (WHO, Regional Office for Africa, 2019). Our study highlights discourses similarly contained in the social construction of disease, enhancing understandings about how epidemics are represented in the public sphere, and the findings suggest that media use alternative discourses that could bring social cohesion, social action, and organised medical management, rather than disease management being disabled by fear and conspiracy.

## Conclusion

This study illustrated the emotive impact that particular cultural representations of Ebola have in newspaper reports. The consensus among the press reports was that South Africa was not at risk, but the discourses have implications. First, reflected in this study through the threat to humanity discourse, Africa’s deviation from Western norms was implied to be the cause of Ebola, and this upholds colonial hegemony. Second, the discourse of a *human* criminal who stalks victims seems more distinctive of South African news reportage compared to discourses in other studies’ findings that relied instead on images of a predatory *microorganism*. Crime headlines in South Africa are not uncommon, and the Ebola reportage deployed similar crime rhetoric. The more common image in other studies about diseases (e.g., AIDS, the plague, tuberculosis) was the waging of a war against an intruder. Ebola, in our research findings, was represented not as a mere intruder but as a human predator. This shift could be considered within the context of individualisation of health risks rather than nationalistic protection because globalisation has removed borders; and readers, rather than nations that can wage war, are allocated responsibility for their health risks. The news reader is incited to be on the lookout for the lurking predator. In the 21st century and in a country at peace, crime against an individual elicits personalised fear, in contrast to the older image of a war that threatens national security. The fear that media incites only occurs because of flaws in health-risk communication, as well as in ‘our fundamentally flawed human psychology . . . The real tragedy of this failure is in the consequences of that fear reaction, both intended and unintended’ (James, 2014, p. 465). Even if fear discourse could help speed up medical research, the predatory discourse, according to Weldon (2001b), is dangerous globally because it reveals an unsophisticated understanding about humans’ biological relationship with the world through their environmental and scientific intrusions. Poor disease control and medical malpractice spread the disease rather than an anthropomorphised entity (Weldon, 2001a), or virulence (Brown et al., 2015; Weldon, 2001b).
